# Rare and lethal complication of barium enema intravasation

**DOI:** 10.1259/bjrcr.20180017

**Published:** 2018-05-24

**Authors:** Guo Hou Loo, Farah Marzuki, Fitjerald Henry

**Affiliations:** 1 Department of General Surgery, Selayang Hospital, Lebuhraya Selayang Kepong, Batu Caves, Selangor, Malaysia; 2 Department of General Surgery, Tengku Ampuan Rahimah Hospital, Jalan Langat, Klang Selangor, Malaysia

## Abstract

Barium enema investigation is regarded as a safe investigative procedure. Rarely, it may cause complications such as colonic perforation and barium intravasation. Barium intravasation may be caused by the inadvertent introduction of the catheter into the vagina, thereby into the vaginal venous plexus. It may also occur through mechanical colonic perforation with the catheter, or via diseased bowel conditions. This may lead to complications such as liver microabscesses, massive pulmonary embolism, hypovolemic shock, disseminated intravascular coagulopathy and even sudden death. With that in mind, we would like to report an interesting case of barium intravasation into the portal venous system via the vagina venous plexus. The patient experienced abdominal discomfort with mild per vaginal bleed and went into disseminated intravascular coagulopathy. She received supportive management and she made a full recovery.

## Case Report

**Figure 1.**
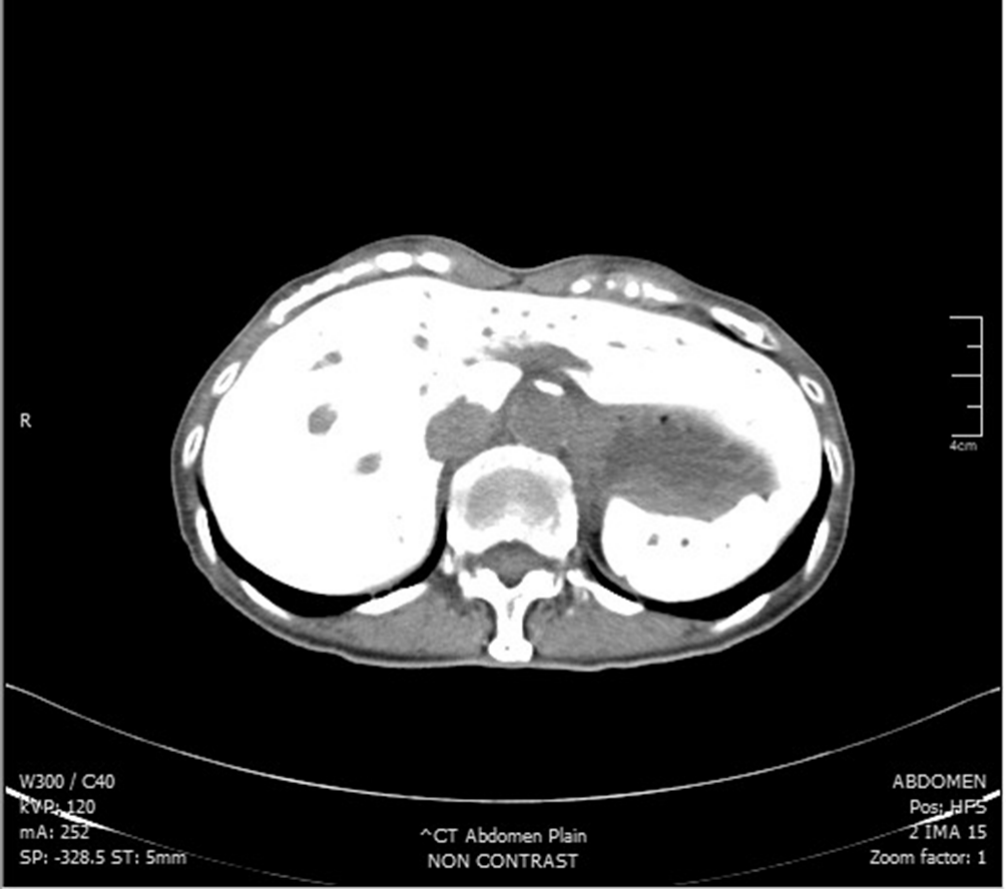
Plain CT abdomen showing barium within the liver and spleen.

**Figure 2.**
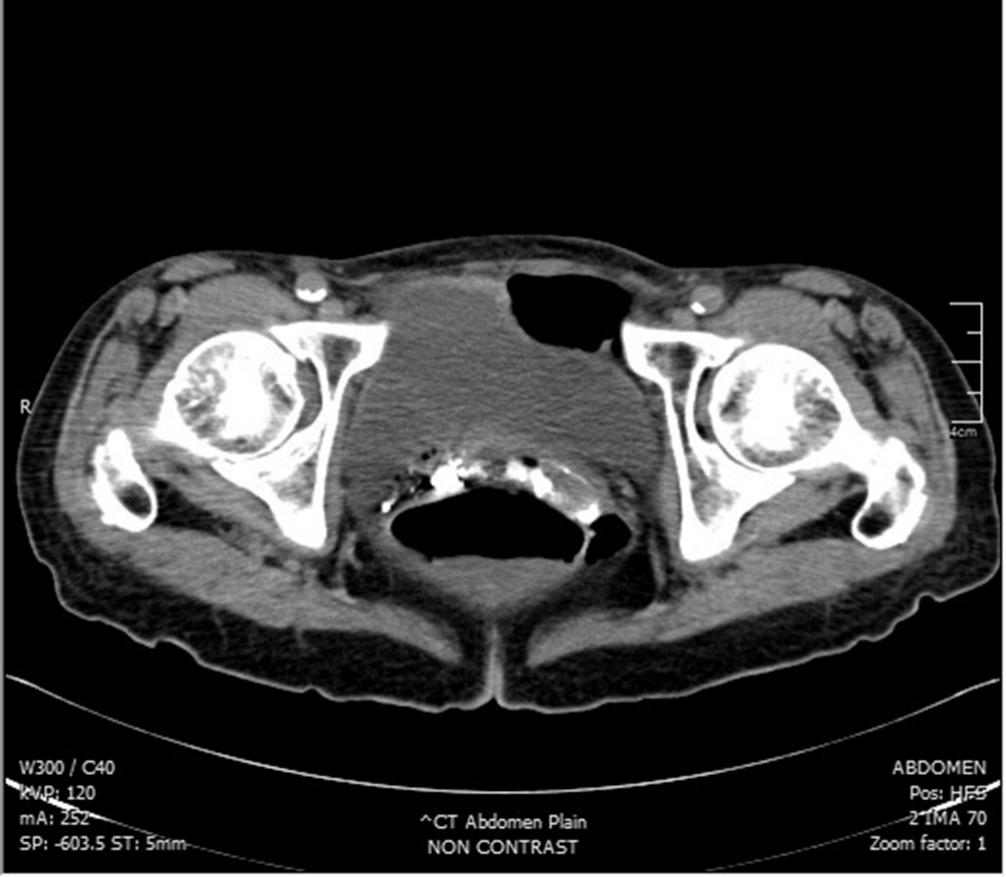
Plain CT showing barium contrast within the uterus and pouch of Douglas. Minimal free fluid and air can also be visualised in the pouch of Douglas.

 A 72-year-old lady was being investigated at our clinic for chronic abdominal pain. She has a history of hypertension. A diagnostic colonoscopy was attempted but due to tight angulation at the splenic flexure, it was abandoned and she was scheduled for a barium enema. Barium enema examination was carried out using a balloon catheter which was placed and inserted by an experienced radiographer. Approximately 100 ml of 60% w/v concentration of barium sulphate was instilled and screening commenced. However, the contrast was seen outside the rectum during screening. The procedure was immediately halted and the catheter removed. The patient complained of mild abdominal discomfort and clinically she was tachycardic with minimal per vaginal bleeding. We proceeded with a plain CT scan of the abdomen and pelvis, which revealed contrast within the liver, spleen Figure 1, uterus, and the pouch of Douglas Figure 2. She was promptly sent to the Emergency Department for further management. Upon clinical assessment, she appeared to be comfortable at rest but was still tachycardic with a pulse rate of 120 bpm. Abdominal examination was unremarkable. Vaginal examination by the gynaecologist revealed an atrophic vagina. No active bleeding was seen. Initial investigations revealed metabolic acidosis (pH 7.238, Bicarbonate 18.3), leukopenia (White cell count 880 ul^−^^1^), mild thrombocytopenia (136,000 ul^−^^1^), raised serum lactate (5.26), deranged coagulation profile [Prothrombin time 32.3, international normalised ratio 3.1, Activated Partial Thromboplastin Time (APTT)161.8]. Her liver enzymes were not raised. She was transferred to the intensive care unit, where supportive treatment was instituted. The patient developed disseminated intravascular coagulopathy which responded to transfusion of fresh frozen plasma and platelets. After 3 days in the intensive care, she returned to the ward. She developed respiratory distress in ward and CTPA was done which ruled out pulmonary embolism. She was treated for pneumonia which responded to intravenous antibiotics. Further prospective review of her plain CT confirmed the presence of contrast in the pelvic veins, consistent with intravasation of barium sulphate from the vagina. She made a full recovery and was discharged.

## Discussion

 Barium enema is usually considered a safe investigation, and the most common serious complication is bowel perforation, which occurs in 0.004–0.04% of cases.^1^ The most fearsome complication, although rare, is barium intravasation, which leads to massive pulmonary embolism, and sudden death. The mortality rate of this complication has been reported to be as high as 67%.^2^ It usually occurs in an elderly female, with a mean age of 60 years. The likely contributing factors include deficient perineal musculature, vaginal atrophy, patient confusion, and the use of balloon catheter.^2^ Other risk factors include obesity, previous colon surgeries, high filling pressure, colonic/vaginal perforation and severe chronic illness.^2^ For our patient, she is a thin built, nulliparous elderly lady, with possible deficient perineal musculature and atrophic vagina, which contributed to this complication. Barium intravasation via lower rectum or vagina usually enters the systemic circulation through the internal iliac veins and then into the lungs where it causes occlusion to the pulmonary circulation.^1^ It may also enter the portal circulation via upper rectum and colonic vessels.^1^ In this patient, it is evident that she has intravasation into the vagina. It is possible that as the vaginal venous plexus has communication with the vesicle and hemorrhoidal plexus, barium then enters the vaginal venous plexus and thus into the portal circulation via this pathway.

**Figure 3.**
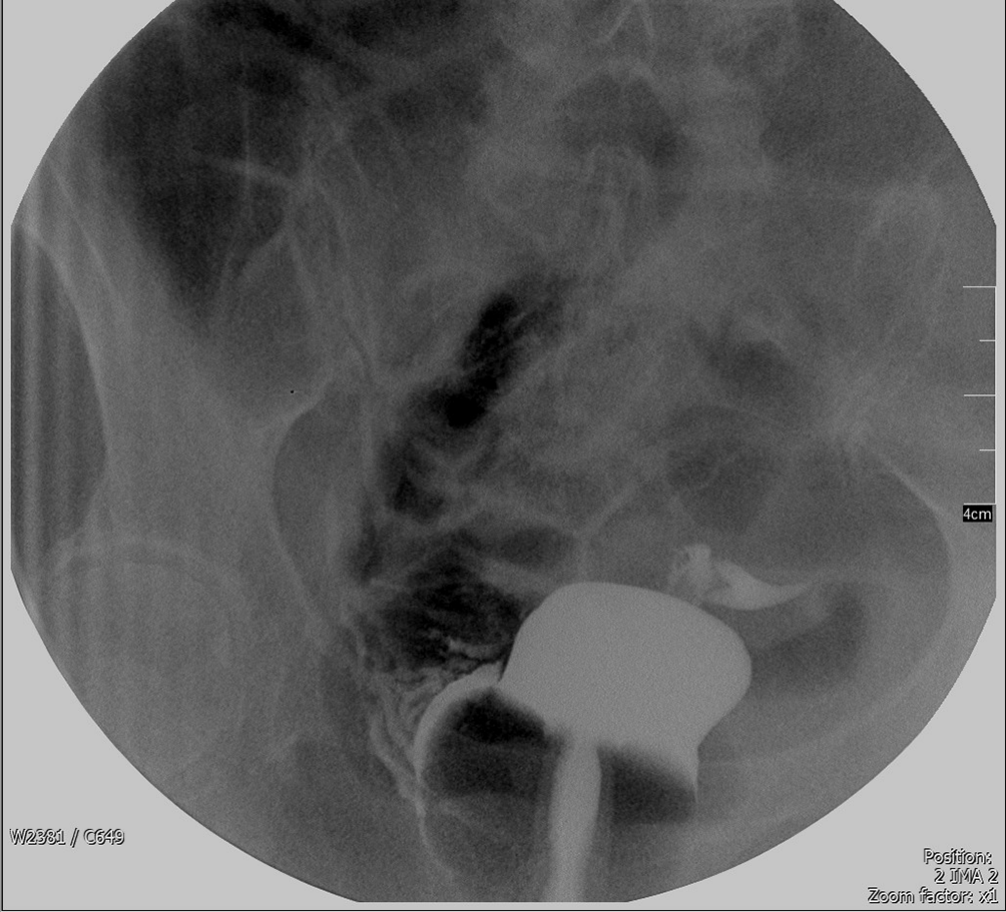
Vaginal intubation with rectal balloon catheter–anteroposterior view.

Clinical features of barium intravasation vary widely. The patient can be asymptomatic, having slight abdominal pain or per vaginal bleeding, or may even present with full-blown hypovolemic shock, disseminated intravascular coagulation and sudden death.^3^ Most deaths are attributed to massive pulmonary embolism.^4^ As barium intravasation is rarely seen, making a diagnosis might prove challenging. A plain pelvic radiograph usually suffice, with it resembling an uterovenography Figure 3.^5^ A plain CT may be performed,^2^ where it will show barium within the solid organs (liver, spleen) or even the lungs Figures 1 and 2. Blood investigation, if taken promptly, may show marked derangements. Leukopenia, thrombocytopenia, metabolic acidosis, raised serum random lactate, increased prothrombin time and international normalised ratioR) may occur.^1^ Of note, in our patient, she developed disseminated intravascular coagulation but her liver enzymes were normal. Treatment is mainly supportive, with close monitoring in the intensive care unit, and support of the hepatic, renal, and cardiovascular function.^1^ Transfusion of blood products may be required to correct coagulopathy. A broad-spectrum antibiotic is needed to cover for possible infection by gram-negative organism carried by contrast material.^2^ As this is an iatrogenic complication, prevention is of utmost importance. Preventive measures include identifying at-risk patients, correct placement of a rectal catheter by an experienced health personnel, avoiding the use of balloon catheters, and control of the barium insufflation pressure.^4^

## Learning points

1. Due to this potentially lethal complication, clinicians ordering this procedure should be aware of this complication, and if possible, to replace it with safer alternatives, such as hydrosoluble contrast medium.
2. Prompt recognition of barium intravasation from both clinical symptoms and investigations as described is needed as swift intervention is necessary to prevent further complications.
3. Utmost importance should be placed on preventing such a complication. The use of balloon catheters should be reduced, and confirmation of placement should be done prior to barium insuffulation. It has been suggested that placement of catheter may be confirmed by insuffulating air or minimal barium contrast and screening at the start of procedure.
